# Synthesis and Photopatterning of Synthetic Thiol-Norbornene Hydrogels

**DOI:** 10.3390/gels10030164

**Published:** 2024-02-23

**Authors:** Umu S. Jalloh, Arielle Gsell, Kirstene A. Gultian, James MacAulay, Abigail Madden, Jillian Smith, Luke Siri, Sebastián L. Vega

**Affiliations:** 1 Department of Biomedical Engineering, Rowan University, Glassboro, NJ 08028, USA; jalloh79@students.rowan.edu (U.S.J.); kirstenegiddings@gmail.com (K.A.G.); macaul14@students.rowan.edu (J.M.); smithjillian428@gmail.com (J.S.); sirilu12@students.rowan.edu (L.S.); 2 Department of Orthopaedic Surgery, Cooper Medical School of Rowan University, Camden, NJ 08103, USA

**Keywords:** synthetic hydrogels, 8-arm PEG, thiol-norbornene, photopatterning

## Abstract

Hydrogels are a class of soft biomaterials and the material of choice for a myriad of biomedical applications due to their biocompatibility and highly tunable mechanical and biochemical properties. Specifically, light-mediated thiol-norbornene click reactions between norbornene-modified macromers and di-thiolated crosslinkers can be used to form base hydrogels amenable to spatial biochemical modifications via subsequent light reactions between pendant norbornenes in the hydrogel network and thiolated peptides. Macromers derived from natural sources (e.g., hyaluronic acid, gelatin, alginate) can cause off-target cell signaling, and this has motivated the use of synthetic macromers such as poly(ethylene glycol) (PEG). In this study, commercially available 8-arm norbornene-modified PEG (PEG-Nor) macromers were reacted with di-thiolated crosslinkers (dithiothreitol, DTT) to form synthetic hydrogels. By varying the PEG-Nor weight percent or DTT concentration, hydrogels with a stiffness range of 3.3 kPa–31.3 kPa were formed. Pendant norbornene groups in these hydrogels were used for secondary reactions to either increase hydrogel stiffness (by reacting with DTT) or to tether mono-thiolated peptides to the hydrogel network. Peptide functionalization has no effect on bulk hydrogel mechanics, and this confirms that mechanical and biochemical signals can be independently controlled. Using photomasks, thiolated peptides can also be photopatterned onto base hydrogels, and mesenchymal stem cells (MSCs) attach and spread on RGD-functionalized PEG-Nor hydrogels. MSCs encapsulated in PEG-Nor hydrogels are also highly viable, demonstrating the ability of this platform to form biocompatible hydrogels for 2D and 3D cell culture with user-defined mechanical and biochemical properties.

## 1. Introduction

Hydrogels are polymeric three-dimensional scaffolds that have emerged as versatile materials with promising applications in tissue engineering. Serving as biocompatible substrates that closely mimic the native extracellular matrix (ECM), hydrogels form a microenvironment that is conducive to cell growth and proliferation [1,2,3,4]. Beyond their structural support, hydrogels can influence cell fate through their mechanical and bioactive properties [5,6,7]. Specifically, hydrogel stiffness has been found to affect cellular spreading and differentiation [8,9,10]. Furthermore, the incorporation of molecules such as proteins and peptides introduce biological signals that guide cell differentiation. The adaptability of these cues within hydrogels provides researchers with the capability to tailor cellular responses, rendering hydrogels an attractive platform for precise and targeted approaches to tissue engineering.

Click chemistry reactions have emerged as a powerful tool for forming hydrogels, offering high selectivity and yield in aqueous settings [11]. These reactions, unlike physically crosslinked hydrogels, establish strong covalent bonds between macromers and crosslinkers, resulting in improved mechanical properties and stability [12]. The versatility of click chemistry encompasses several biorthogonal click reactions including thiol-norbornene Michael additions [13], tetrazine-norbornene [14], and azide-alkyne reactions. These systems facilitate efficient hydrogel synthesis and modification by selectively reacting with specific functional groups. Natural polymers, such as hyaluronic acid, gelatin, and alginate, are commonly used macromers for hydrogels formed using thiol-norbornene click chemistry reactions [15]. However, cell surface receptors can interact with naturally derived macromers. This limits the ability to adequately study the role of specific material properties on cell behavior, and it could also introduce off-target cell signaling. These limitations have prompted the development of thiol-norbornene hydrogels formed with synthetic macromers, such as polyacrylamide and polyethylene glycol (PEG) [15,16,17].

PEG-based complexes can be modified with norbornene functional groups at the terminal of each PEG-arm (PEG-Nor). Thiol-norbornene click chemistry reactions between PEG-Nor macromers and di-thiol crosslinkers rapidly form covalent bonds, resulting in the formation of a synthetic hydrogel network. Using this light-mediated hydrogel photopolymerization scheme, PEG-Nor hydrogel properties (including stiffness, swelling, degradation rate, and biofunctionalization) can be readily tuned [18,19,20]. Additionally, PEG-Nor hydrogel interactions with cells exhibit no off-target cell signaling, and cells atop and within PEG-Nor hydrogels are highly biocompatible [21]. These qualities render PEG-Nor hydrogels a suitable scaffold for tissue engineering and regenerative medicine applications [13,22,23,24,25,26].

Photopatterning base hydrogels with crosslinkers or bioactive molecules provides an in-situ approach to modifying the microenvironment with spatiotemporal control. Photopatterning versatility has been demonstrated through ice-templating [27], photolithography [28,29,30,31,32], and laser lithography [33,34]. By leveraging the UV light-dependent nature and selectivity of thiol-norbornene reactions towards available functional groups, the initial hydrogels formed using this chemistry can be photopatterned through a secondary reaction that targets available norbornene moieties on the macromer.

This study reports the use of commercially available 8-arm PEG-Nor macromers and di-thiolated dithiothreitol (DTT) crosslinkers to form biocompatible hydrogels amenable to sequential photopatterning with mono-thiolated peptides. The initial degree of crosslinking within the hydrogels was controlled to form a network with distinct mechanical properties while retaining available norbornene groups for secondary reactions with thiol-containing molecules. These hydrogels support 2D and 3D cell culture, and by utilizing the large number of reactive handles in 8-arm PEG-Nor hydrogels, multiple signals were precisely patterned in a spatially and temporally controlled manner throughout the scaffold.

## 2. Results and Discussion

### 2.1. PEG-Nor Hydrogel Mechanics Are Highly Tunable and Base Hydrogels Are Amenable to Secondary Modifications with Thiolated Molecules

**Figure 1:**
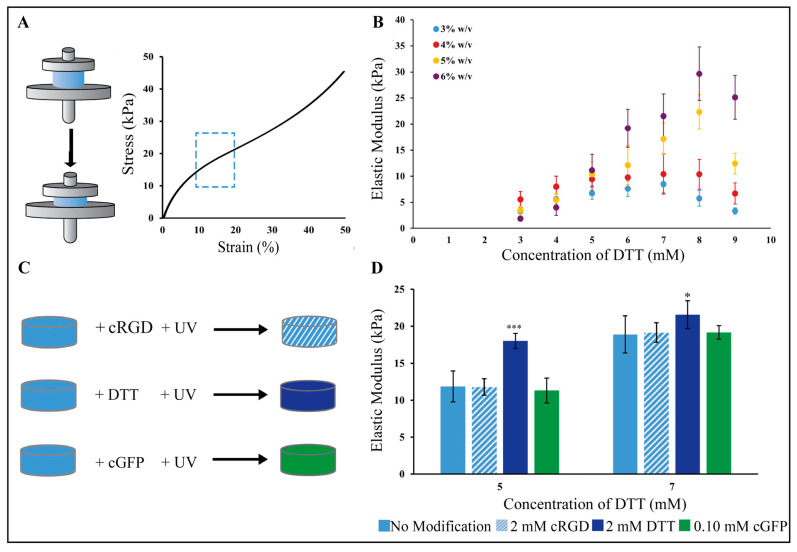
Mechanical characterization of PEG-Nor hydrogels. (**A**) Compressive mechanical testing is performed to measure the elastic modulus, which is calculated as the slope between 10 and 20% strain in a stress–strain curve (blue dashed box). (**B**) Elastic moduli as a function of DTT concentration for 3, 4, 5 and 6 wt% PEG-Nor hydrogel compositions. (**C**) Schematic shows an experimental design for secondary reactions of base PEG-Nor hydrogels with mono-thiolated (cRGD, cGFP) and di-thiolated molecules (DTT). (**D**) Bar graph shows elastic moduli after secondary reactions in 5 wt% PEG-Nor hydrogels with 5 and 7 mM DTT concentration. Bar graphs and scatter plot dots represent the mean and error bars represent standard deviation, * *p* < 0.05, *** *p* < 0.001.

PEG-Nor gels were synthesized using thiol-norbornene click chemistry by reacting norbornene groups in 8-arm PEG-Nor with di-thiolated crosslinkers (DTT) using a photoinitiator and UV light. The photopolymerization of base hydrogels consists of norbornene groups in PEG-Nor reacting with thiols in DTT crosslinkers, so it is expected that a range of mechanics can be achieved by adjusting DTT crosslinker concentration at a constant PEG-Nor wt%. Since there will be more available norbornene groups at higher PEG-Nor wt%, it is also expected that a higher range of mechanics can be achieved with more PEG-Nor present in the hydrogel network. Indeed, Gramlich et al. showed that, by varying macromer content and DTT concentration, hyaluronic acid thiol-norbornene hydrogels with a broad range of mechanics can be synthesized [13]. Compression testing was performed one day after hydrogel formation to determine the elastic moduli of hydrogels formed (Figure 1A). Mechanical testing of unconfined hydrogels in solution one day post-formation allowed for hydrogel swelling to reach a steady-state, and this is a commonly used time-point for compression testing [6,7,13,35].The elastic moduli of base hydrogels were controlled by varying the concentration of DTT in the hydrogel solution at a constant PEG-Nor weight percent (Figure 1B). For 3 wt% PEG-Nor hydrogels, the elastic modulus ranged from 3.3 kPa to 5.7 kPa, and for 4 wt% PEG-Nor hydrogels, the elastic modulus peaked at 11.8 kPa when formed with DTT at a 7 mM concentration. For 5 wt% PEG-Nor hydrogels, the elastic modulus also increased in a crosslinker concentration-dependent manner, reaching a maximum elastic modulus of 22 kPa when formed with DTT at an 8 mM concentration. The elastic modulus of 6 wt% PEG-Nor hydrogels also peaked at an 8 mM DTT concentration (31.3 kPa). Upon reaching a maximum elastic modulus, the stiffness of all formulations started to decrease with increasing DTT concentration. It is expected that this phenomenon is due to the presence of too many thiols, resulting in di-thiolated DTT only binding to one norbornene in PEG-Nor, which hinders the ability for crosslinks to form, as this requires each thiol in the crosslinker to bind to a separate PEG-Nor molecule.

Following PEG-Nor hydrogel formation, a secondary photopolymerization reaction was performed to either increase hydrogel stiffness or to incorporate thiolated peptides in situ. Secondary hydrogel modifications were evaluated on initial gel compositions of 5 wt% PEG-Nor with DTT concentrations of 5 and 7 mM. These concentrations ensured a sufficient crosslink density to establish a hydrogel network while leaving unreacted norbornene moieties available for secondary reactions. The secondary reactions were conducted by subjecting 5 wt% base PEG-Nor hydrogels (containing either 5 mM or 7 mM DTT) to a PBS solution containing photoinitiator I2959 and either 2 mM cRGD, 2 mM DTT, or 0.10 mM cGFP for 10 min, followed by UV irradiation (Figure 1C). The elastic modulus of the modified hydrogels was measured to determine the effects of the secondary molecules on stiffness. Hydrogels functionalized with cRGD and cGFP resulted in non-significant changes in elastic modulus compared to base hydrogels (Figure 1D). In contrast, using DTT as a secondary reaction molecule resulted in a significant increase in elastic modulus for PEG-Nor hydrogels formed with 5 mM (*p* = 0.0001) and 7 mM (*p* = 0.0263) DTT. Mono-thiol peptides, including cRGD and cGFP, exhibit the capability to form only a single bond with available norbornene binding sites on the hydrogel network, lacking the ability to establish new crosslinks within the hydrogel network, and thereby they do not modify the overall elastic modulus. In contrast, di-thiol molecules like DTT can facilitate secondary crosslinking, resulting in an increase in bulk hydrogel mechanical properties. Taken together, this demonstrates that base PEG-Nor hydrogels can be modified with bioactive peptides that have no impact on mechanics, and they can also be modified with additional di-thiolated crosslinkers to increase bulk hydrogel mechanics in situ.

### 2.2. PEG-Nor Hydrogels Can Be Photopatterned with Multiple Mono-Thiolated Peptides

**Figure 2:**
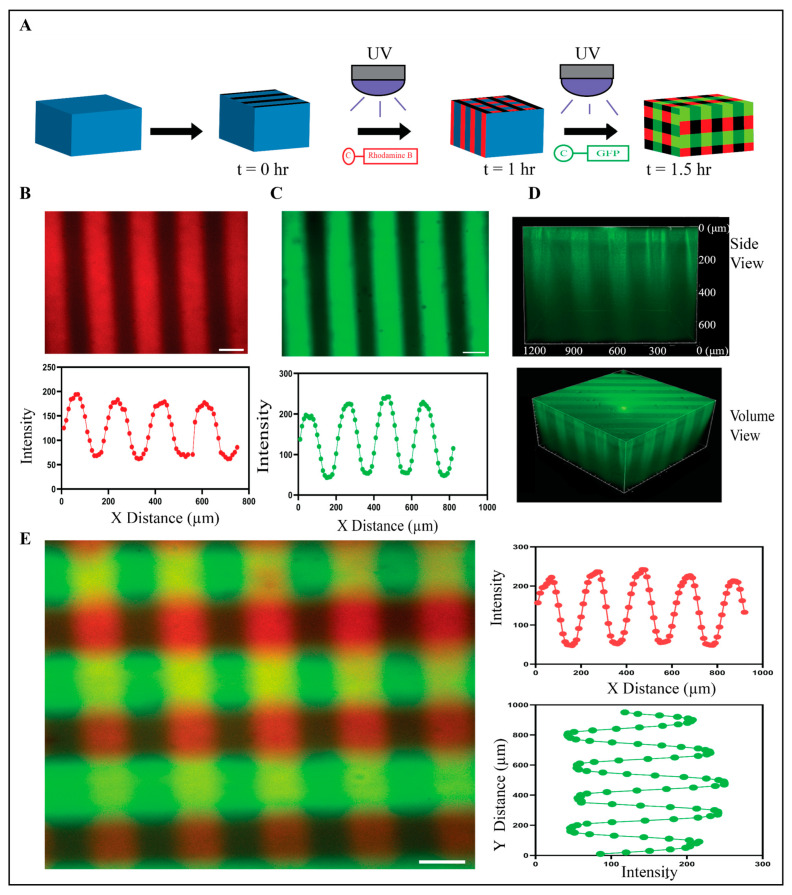
Photopatterning of mono-thiolated peptides onto PEG-Nor hydrogels. (**A**) Schematic shows photopatterning process for one peptide (cRhodamine shown) and for two peptides (cRhodamine followed by cGFP). (**B**) Representative confocal image and plot profile of PEG-Nor hydrogel photopatterned with cRhodamine. (**C**) Representative confocal image and plot profile of PEG-Nor hydrogel photopatterned with cGFP. (**D**) Side view and volume view of photopatterned PEG-Nor hydrogel with cGFP. (**E**) Representative confocal image and intensity plot profiles of sequential photopatterning of PEG-Nor hydrogel with vertical cRhodamine and horizontal cGFP stripes. Scale bars: (**B**,**C**,**E**) = 100 μm.

To evaluate the ability to photopattern base PEG-Nor hydrogels, mono-thiolated rhodamine B (cRhodamine), and mono-thiolated 5(6)-carboxyfluorescein (cGFP) peptides were used (Figure 2A). PEG-Nor hydrogels (5 wt%, 5 mM DTT) were incubated in cRhodamine (100 µM) for 30 min and then a photomask consisting of vertical stripes (100 µm thick, 100 µm spacing) was applied during UV irradiation (10 mW/cm^2^, 60 s), resulting in cRhodamine covalently attaching to the hydrogel only in regions exposed to light (Figure 2B). The same process was performed on a separate set of base hydrogels, with cGFP, resulting in the tethering of cGFP peptides matching the photomask used for peptide patterning (Figure 2C). Representative volumetric and lateral views show that the peptide patterning extends into the hydrogel in the z-direction (Figure 2D), which is useful for 3D cell culture. To evaluate the ability to photopattern multiple peptides at user-defined time-points, base hydrogels were photopatterned with cRhodamine using a vertically striped mask 60 min after initial gelation. Thirty minutes after that, a tertiary photopatterning step with cGFP using a horizontally striped mask was performed (Figure 2E). These photopatterning studies show that base PEG-Nor hydrogels can be photopolymerized via secondary and tertiary thiol-norbornene reactions, resulting in the addition of multiple peptides covalently bound in distinct patterns at user-defined time-points.

### 2.3. PEG-Nor Hydrogels Functionalized with Thiolated RGD Peptides Support 2D Cell Culture

**Figure 3:**
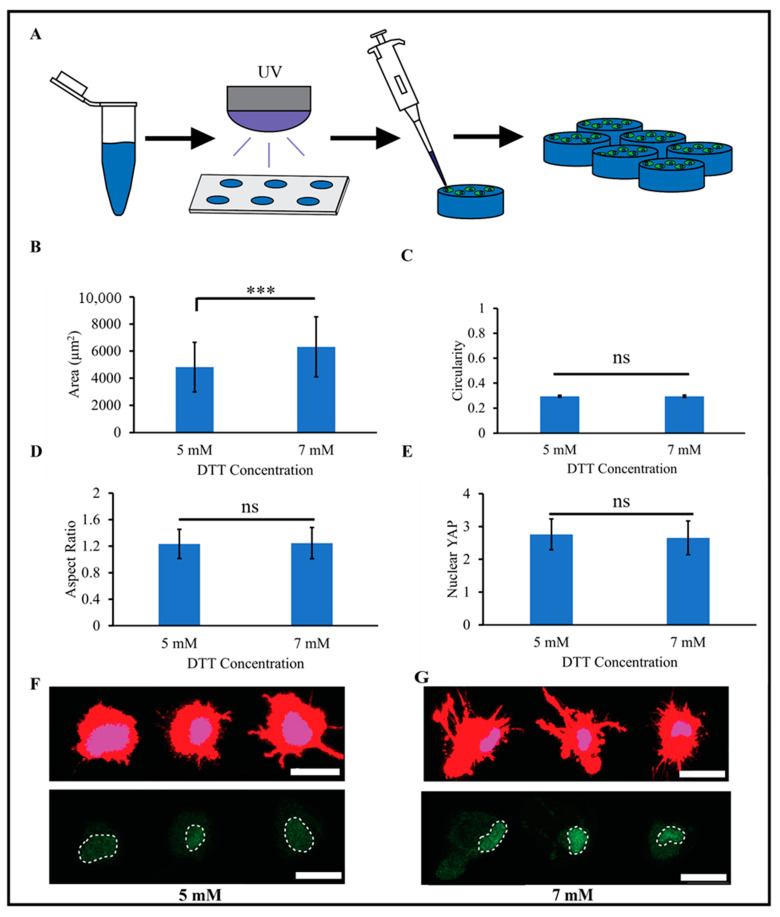
MSCs attach and are mechanically active on RGD-functionalized PEG-Nor hydrogels. (**A**) Schematic of the experimental design for 2D MSC PEG-Nor studies. 2D morphological analysis of cell (**B**) area, (**C**) circularity, and (**D**) aspect ratio of MSCs on RGD-functionalized PEG-Nor hydrogels formed with 5 mM or 7 mM DTT crosslinker concentrations. (**E**) Quantification of nuclear YAP localization of MSCs on RGD-functionalized PEG-Nor hydrogels formed with 5 mM or 7 mM DTT crosslinker concentrations. Representative images of single MSCs stained for cytoskeletal actin (red), nuclei (blue), and YAP (green) on top of (**F**) 5 mM DTT and (**G**) 7 mM DTT RGD-functionalized PEG-Nor hydrogels (dashed white lines denote nuclear outlines). Bars represent the mean and error bars represent standard deviation, *** *p* < 0.001, while ns indicates not statistically significant. Scale bars: (**F**,**G**) = 25 μm.

PEG is a non-fouling polymer that is resistant to protein adsorption, and adherent cells are unable to attach to untreated PEG surfaces [36]. The inclusion of adhesive moieties is needed for cellular adhesion, and Nguyen et al. demonstrated that HUVECs (human umbilical vein endothelial cells) cultured on top of PEG-Nor hydrogels functionalized with 2 mM cRGD adhered and indicated highly viability in comparison to PEG-Nor hydrogels without cRGD [37]. To evaluate the use of PEG-Nor hydrogels as a 2D cell culture system, 5 wt% PEG-Nor hydrogels were synthesized on silicone molds with two concentrations of DTT (5 mM or 7 mM) and with mono-thiolated RGD (cRGD, 2 mM) (Figure 3A). Human mesenchymal stem cells (MSCs, 10,000 cells/cm^2^) were then cultured atop the hydrogels, and their adhesion, morphology, and matrix mechanosensing were evaluated. MSCs readily attached on cRGD-functionalized PEG-Nor hydrogels, demonstrating that, not only do mono-thiolated peptides covalently bind to PEG-Nor hydrogels, but they also retain their bioactivity. MSCs on the 5 mM DTT PEG-Nor hydrogels were slightly smaller (4800 μm^2^ ± 1800 μm^2^) than those on PEG-Nor hydrogels formed with 7 mM DTT (6300 μm^2^ ± 2200 μm^2^) (Figure 3B). The elastic moduli of PEG-Nor hydrogels formed with 5 mM and 7 mM DTT were 9.9 kPa and 17.2 kPa, respectively, and MSC area increases with increasing stiffness on 2D materials [38].

An analysis of other morphological metrics showed no statistically significant differences between the two PEG-Nor groups. Circularity is a measure of roundness ranging from 0 (line) to 1 (perfect circle), and the values for both groups averaged 0.30 ± 0.01 (Figure 3C). Although circularity was low, the aspect ratio was only about 20% above unity (Figure 3D), suggesting that the cells in both groups spread isotropically with pronounced protrusions. The extent of nuclear YAP (Yes-associated protein) localization is strongly correlated to cellular matrix mechanosensing [39], and nuclear YAP values were insignificant between groups, averaging 2.70 ± 0.50 for both groups (Figure 3E). Overall, MSC morphology and nuclear YAP localization are highly similar between MSCs on PEG-Nor hydrogels formed with 5 mM DTT (Figure 3F) and 7 mM DTT (Figure 3G) crosslinkers, and these findings show that these hydrogels support 2D cell culture. Human bone marrow stem cells cultured on hydrogels of comparable stiffness (~12–24 kPa) proliferate and feature enhanced cytoskeletal formation and cell spreading [9], which was also observed in this study. MSCs on stiff materials also preferentially differentiate into osteoblasts [38,40,41], and 2D differentiation studies of MSCs on the PEG-Nor hydrogels presented warrant further investigation.

### 2.4. MSCs Are Spherical and Highly Viable in 3D PEG-Nor Hydrogels

**Figure 4:**
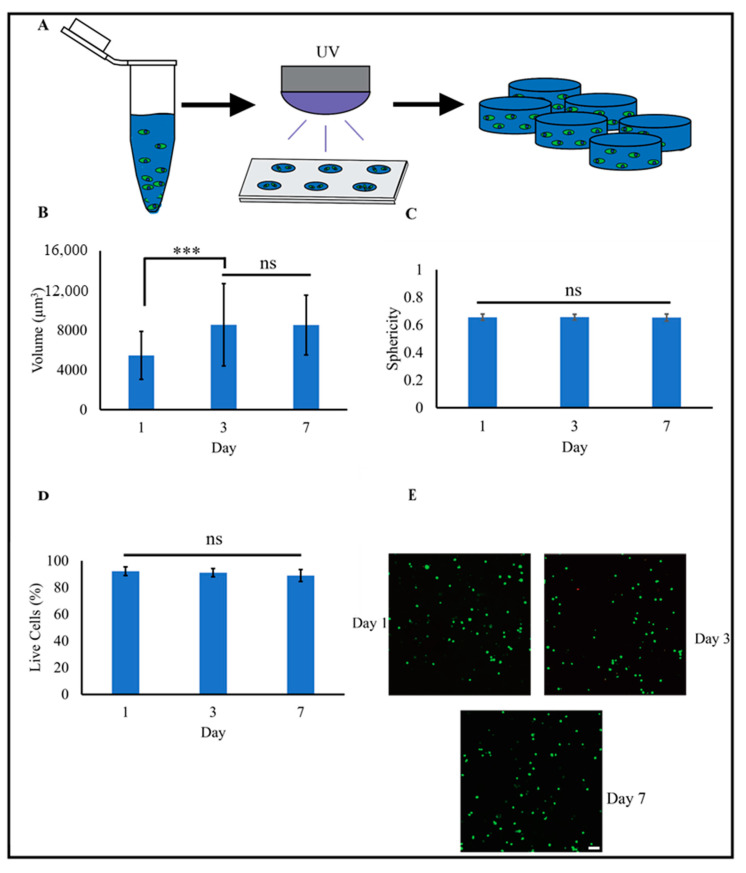
MSCs encapsulated in PEG-Nor hydrogels are round and highly viable. (**A**) Schematic of forming PEG-Nor hydrogels with encapsulated MSCs. 3D morphological analysis of cell (**B**) volume and (**C**) sphericity after 1, 3, and 7 days in culture. (**D**) Percentage of live MSCs after 1, 3, and 7 days in culture, and (**E**) representative confocal images of viable MSCs encapsulated in PEG-Nor hydrogels after 1, 3, and 7 days in culture. Bars represent the mean, while error bars represent standard deviation, *** *p* < 0.001, and ns indicates not statistically significant. Scale bar: (**E**) = 100 μm.

To evaluate 3D biocompatibility, MSCs were encapsulated (1 million cells/mL) in PEG-Nor hydrogels (5 wt%) formed with 5 mM DTT (Figure 4A). Notably, 3D morphological analyses of individual MSCs showed a significant difference in cell volume between 1- and 3 days; however, no significant differences were observed after 3- or 7 days in culture (Figure 4B). The observed increase in cell volume between day one and day three could be attributed to hydrogel swelling shortly after the hydrogel is formed. As the hydrogel swells within the first few days, it can create more space within the matrix [42,43], allowing cells to expand. No significant differences were observed in sphericity (values range from 0 to 1, where 1 is a perfect sphere), and values were 0.65 and 0.66 for MSCs encapsulated in 3D PEG-Nor hydrogels after 1 and 7 days, respectively (Figure 4C). High sphericity is expected in this study, as encapsulated cells lack the ability to remodel the local hydrogel environment. To promote 3D cell spreading, which is necessary for increased cellular matrix mechanosensing and favors osteogenic differentiation [44,45], di-thiolated enzymatically degradable crosslinker peptides can be used in lieu of DTT, as carried out in previous studies [45]. A live–dead analysis also showed high cell viability, with over 90% of cells remaining viable after at least seven days in culture (Figure 4D).

## 3. Conclusions

This study describes a simple method to create synthetic hydrogels using commercially available 8-arm PEG-Nor macromers and DTT crosslinkers. By varying PEG-Nor macromer and DTT crosslinker concentration, base hydrogels with a wide range of elastic moduli values can be synthesized. The pendant norbornenes on these hydrogels can then be used to spatially tether mono-thiolated peptides via subsequent light-mediated thiol-norbornene reactions using photomasks. This study demonstrates that thiolated fluorescent peptides and thiolated RGD can be covalently bound to PEG-Nor hydrogels, and the same scheme can also be applied to other peptides. For instance, HAVDI is the mimetic peptide sequence of *N*-cadherin cell–cell interactions, and it has been shown to attenuate 2D cell-matrix mechanosensing. Additionally, it can also promote 3D chondrogenic differentiation of encapsulated MSCs [46,47]. As another tissue engineering application, the DWIVA sequence mimics the osteogenic domains of bone morphogenetic protein-2, and MSCs encapsulated in DWIVA-functionalized peptides differentiate into osteoblasts in vitro [48], while injectable hydrogels with this BMP-2 mimetic peptide induce nascent bone formation in the rat femurs in vivo [48,49].

## 4. Materials and Methods

### 4.1. Materials

For this work, 8-arm PEG-norbornene (MW 20 kg/mol) was purchased from JenKem Technology (Plano, TX, USA). DTT (dithiothreitol), photoinitiator Irgacure 2959, and Triton X-100 were purchased from Sigma-Aldrich (St. Louis, MO, USA). Mono-thiolated Arginine-Glycine-Aspartic Acid (cRGD, sequence: GCGYGRGDSPG) and mono-thiolated 5(6)-carboxyfluorescein (cGFP, sequence: GCDDD-5(6)-carboxyfluorescein) were purchased from GenScript (Piscataway, NJ, USA). Mono-thiolated rhodamine B (cRhodamine, sequence: GCDDD-rhodamine B) was synthesized using an in-house solid state peptide synthesizer (Liberty Blue, CEM, Matthews, NC, USA). Peptide purity was confirmed with MALDI-TOF spectrometry. PBS (phosphate-buffered saline), formalin, ethidium homodimer III, Hoechst, BSA (bovine serum albumin), and penicillin-streptomycin were purchased from VWR (Radnor, PA, USA). Additionally, α-MEM, FBS (fetal bovine serum), and calcein AM were purchased from Thermo Fisher Scientific (Waltham, MA, USA). Human MSCs used for cell culture were purchased from Lonza Bioscience (Walkersville, MD, USA).

### 4.2. Base PEG-Nor Hydrogel Synthesis and Mechanical Testing

**Figure 5:**
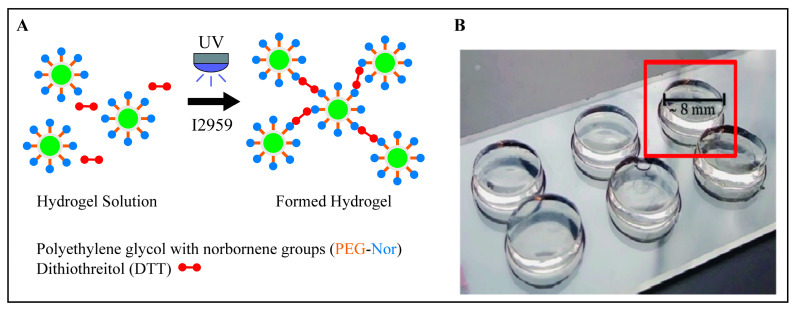
Synthesis of PEG-Nor hydrogels using thiol-norbornene click chemistry. (**A**) Solution containing 8-arm PEG-Nor macromer, dithiol crosslinker DTT, and photoinitiator I2959 in the presence of UV light reacts to form PEG-Nor hydrogels. (**B**) Cylindrically formed 3D PEG-Nor hydrogels with 8 mm diameter and 2 mm height.

Hydrogels were prepared by dissolving 8-arm PEG-Nor macromer in PBS at 3, 4, 5, or 6 wt% with varying amounts of DTT and 0.05 wt% I2959 (Figure 5A). By varying the DTT concentration at a fixed PEG-Nor macromer wt%, a range of bulk base hydrogel mechanics were achieved, and Figure 1B summarizes elastic moduli values achieved for specific PEG-Nor macromer wt% and DTT concentrations. Pre-hydrogel solutions were vortexed and, after being fully dissolved, 90 µL of the hydrogel solution was added to cylindrical (8 mm diameter, 2 mm height) polydimethylsiloxane (PDMS) molds and irradiated with UV light (320–390 nm, 10 mW/cm^2^, 10 min). Formed hydrogels were removed from the molds (Figure 5B) and placed in cell culture wells containing 1 mL PBS to swell overnight at 37 °C prior to mechanical testing. Elastic moduli were determined using a Shimadzu EZ-SX Mechanical Tester by compressing hydrogels at a constant strain rate of 10% per minute. The elastic modulus was calculated from the slope of the stress–strain curve between 10 and 20% strain.

### 4.3. Secondary Modifications to PEG-Nor Hydrogels

To modify base PEG-Nor hydrogels with mono-thiolated peptides or DTT crosslinkers, PEG-Nor hydrogels (5 wt% PEG-Nor with either 5 mM or 7 mM DTT) were incubated in either cell adhesive cRGD (2 mM) peptide, DTT crosslinker (2 mM), or green fluorescent cGFP (0.10 mM) peptide, in a PBS solution containing 0.05 wt% I2959 for 10 min. The soaked hydrogels contained untethered thiolated molecules which were then covalently bound to the whole hydrogel network by irradiating the samples with UV light (5 mW/cm^2^, 1 min).

To photopattern base PEG-Nor hydrogels with vertical cRhodamine stripes, base PEG-Nor hydrogels (5 wt% PEG-Nor, 5 mM DTT) were incubated in a cRhodamine (100 µM) PBS solution containing 0.05 wt% I2959 for 10 min. A striped photomask (100 µm stripe thickness, 100 µm stripe spacing) was then carefully placed atop a soaked hydrogel containing untethered cRhodamine, and the cRhodamine was covalently bound to the hydrogel network only in regions that allowed UV light (5 mW/cm^2^, 1 min) to pass through. The hydrogel then underwent several PBS washes to remove unbound cRhodamine and was imaged with a Nikon A1R confocal microscope. To photopattern base PEG-Nor hydrogels with vertical green, fluorescent stripes, the same procedure for patterning vertical red fluorescent stripes was followed, but cRhodamine was substituted with cGFP (100 µM). To photopattern base PEG-Nor hydrogels with vertical red fluorescent stripes and horizontal green, fluorescent stripes, the procedure described above was followed sequentially, starting with cRhodamine photopatterning followed by cGFP photopatterning achieved by rotating the same photomask 90 degrees.

### 4.4. Human MSC Culture and 2D PEG-Nor Cell Culture

Human MSCs P4 (Lonza) were expanded in growth medium (α-MEM supplemented with 10% FBS and 1% penicillin/streptomycin). For 2D studies, hydrogels were prepared by dissolving 8-arm PEG-Nor macromer in PBS at 5 wt% with 5 mM or 7 mM DTT, 2 mM cRGD, and 0.05 wt% I2959. The solution was vortexed, and after it was fully dissolved, 90 µL of the hydrogel solution was added to cylindrical (8 mm diameter, 500 µm height) silicone molds and irradiated with UV light (10 mW/cm^2^, 10 min). Hydrogels in the molds were then placed in wells of 24-well plates and underwent two 5 min washes with growth medium, while MSCs were seeded on top at a seeding density of 10,000 cells/cm^2^.

### 4.5. 3D PEG-Nor Cell Culture

For 3D studies, MSCs suspended in growth medium were centrifuged (500 RCF, 5 min) and resuspended in a growth medium solution containing 5 wt% PEG-Nor, 5 mM DTT, 2 mM cRGD, and 0.05 wt% I2959 to achieve a cell concentration of 1 million cells/mL. Hydrogel solutions were then photopolymerized with UV light (10 mW/cm^2^, 10 min), and the newly formed hydrogels were removed from the molds and underwent two successive 5 min washes with growth medium to eliminate free radicals generated during the thiol-norbornene photopolymerization reaction.

### 4.6. Imaging and Image Analysis

For 2D analysis, maximum projection confocal images were analyzed with ImageJ software (version 1.53t). Briefly, binary masks were created from the actin channel using Otsu-based thresholding. The masks were then subjected to the Measure function which automatically computed Area, Circularity, and Aspect Ratio for individual cell masks. Otsu-based thresholding was then used to obtain nuclear masks using the Hoescht channel, and these masks were superimposed onto the YAP channel to calculate the YAP-integrated density (sum of pixel intensities ranging from 0 to 255 per pixel) in the nucleus and in the whole cell. From this data, the ratio between the present cytosolic YAP and nuclear YAP was determined using the following equation:

$$Nuclear\;YAP\;=\;\frac{\frac{Nuclear\;YAP\;Signal}{Nucleus\;Area}}{\frac{Cytosolic\;YAP\;Signal}{Cytosolic\;Area}}$$

To evaluate 3D morphology, samples were fixed, permeabilized, and stained with phalloidin (actin) and Hoescht (nuclei). Using 3D image stacks, the Otsu thresholding technique was used to create masks from the actin and Hoescht channel stacks, and the measure function was used to calculate single-cell volume and sphericity from the 3D cellular masks. Sphericity values ranged from 0 to 1, where 1 was a perfect sphere.

To evaluate viability, MSC-laden hydrogels were cultured for 1, 3, or 7 days, and samples were cultured in a Live/Dead viability working solution (growth medium supplemented with 1:1000 calcein AM and 1:1000 ethidium homodimer) for 30 min prior to imaging on an A1R Nikon confocal microscope. For each MSC-laden PEG-Nor hydrogel sample, image stacks were captured with a step size of 3.2 μm at a height of 100 μm. Maximum projections of the image stacks were then used to count the number of live cells (green), while viability was determined by dividing the total number of viable cells by the total cell count (green living cells plus dead red cells).

### 4.7. Statistical Analysis

All data are from three independent biological experiments. For viability, at least 500 cells were counted per condition, and for 3D morphology analysis, at least 50 cells were quantified per time-point. Statistical analyses were performed with JMP Pro 17. Differences among groups are stated as *p* < 0.01 (*), *p* < 0.001 (***) and as (ns) when differences between groups were not statistically significant.

## Data Availability

The raw data supporting the conclusions of this article will be made available by the authors on request.
